# Lessening Loneliness in Adolescents: A Scoping Review of Interventions Used to Combat Loneliness in Adolescents

**DOI:** 10.1192/bjo.2026.11059

**Published:** 2026-06-30

**Authors:** Ching Stephanie Ho, Christina Victor

**Affiliations:** 1 Brunel Medical School, Brunel University London, London, United Kingdom; 2 Department of Health Sciences, Brunel University London, London, United Kingdom

## Abstract

**Aims:**

Globally, up to 15% of adolescents (ages 10–19) experience loneliness, reportedly harming health. This scoping review systematically explores interventions combating adolescent loneliness worldwide, a topic with limited existing literature.

The aim is to map and characterise existing interventions for adolescent loneliness and evaluate the quality of available evidence.

Preliminary research uncovered systematic reviews and meta-analyses reviewing interventions combating adolescent loneliness worldwide. As multiple interventions were analysed by said systematic reviews and meta-analyses, this led to the hypothesis that a growing body of research on interventions combating adolescent loneliness worldwide exists.

**Methods:**

The research protocol followed Joanna Briggs Institute JBI scoping review guidelines and the Preferred Reporting Items for Systematic Reviews and Meta-Analyses extension for Scoping Reviews (PRISMA-ScR). From April to June 2024, literature searches were conducted on PubMed and five EBSCO host databases, using a search string. Time frame for study inclusion was January 2000 to 23 April 2024. Data extraction and analysis were conducted manually. Quality of systematic reviews and meta-analyses were assessed using A Measurement Tool to Assess Systematic Reviews (AMSTAR).

**Results:**

The literature search uncovered 16 papers. The 9 selected for review included 7 countries (Portugal, the USA, Canada, Israel, China, Australia, and the UK), and encompassed 2 systematic reviews and one meta-analysis rated critically low, low, and moderate quality, respectively. Several studies had small sample sizes, the median being 43.5, and range being 4 to 2966. Intervention types were categorized into group-based (n=37), individual (n=19), or mixed group and individual (n=3). Data were excluded from one systematic review that did not specify intervention type. Thematic analysis revealed 11 out of 16 interventions from a systematic review were classed as high-intensity. 3 studies suggested benefits of high-intensity interventions.

**Conclusion:**

Consistent with the hypothesis, there is a growing body of research oninterventions combating adolescent loneliness worldwide. However, closer analysis of existing research indicates further studies are needed, with larger sample sizes, more diverse demographics and standardised research methods and outcome measures. More high-quality primary studies are needed to produce quality systematic reviews. Considering resources required for high-intensity interventions, future research may focus on health economics and cost-benefit analyses.

This study received no external funding.

